# IL-27 neutralization with and without antibiotics as an approach to prevent and treat neonatal sepsis

**DOI:** 10.1172/jci.insight.204603

**Published:** 2026-07-23

**Authors:** Madhavi Annamanedi, Jessica M. Povroznik, Samantha Arevalo-Marcano, Cory M. Robinson

**Affiliations:** 1 Department of Microbiology, Immunology, and Cell Biology, and; 2 Department of Pediatrics, West Virginia University School of Medicine, Morgantown, West Virginia, USA.; 3 Vaccine Development Center, West Virginia University Health Sciences Center, Morgantown, West Virginia, USA.

**Keywords:** Immunology, Infectious disease, Inflammation, Bacterial infections, Cytokines, Immunotherapy

## Abstract

Neonatal sepsis is a predominant cause of neonatal mortality and long-term morbidity that severely affects preterm and low-birth-weight newborns. Antibiotic resistance and long-term developmental issues associated with neonatal sepsis necessitate finding new and improved treatment options. IL-27 has diverse influences on the immune response, is elevated during the neonatal period compared with adulthood, and continues to rise further during infection. Elevated levels of IL-27 early in life predispose the host to impaired control of the pathogen burden and increased mortality. This study explored the therapeutic potential of IL-27p28 antibody administration to improve treatment outcomes during murine neonatal sepsis. Sepsis was induced by subcutaneous inoculation of K1-encapsulated *E*. *coli*, and the neonatal pups were rescued with IL-27p28 monoclonal antibody. Pups that received prophylactic antibody prior to the infection demonstrated superior bacterial clearance and significant weight gain compared with controls during infection. The combination of subclinical dose of gentamicin along with IL-27p28 antibody administered 2 hours after infection, significantly improved bacterial clearance and glucose homeostasis, with reduced serum levels of IL-6 and TNF-α, vital organ damage, and improved the survival rate of infected pups compared with gentamicin alone. These findings suggest that IL-27p28 antagonization represents a promising therapeutic tool for treatment of neonatal sepsis.

## Abstract 2

Neonates have increased susceptibility to bacterial sepsis and the research described in this work offers insight into IL-27 neutralization as a new treatment strategy.

## Graphical abstract

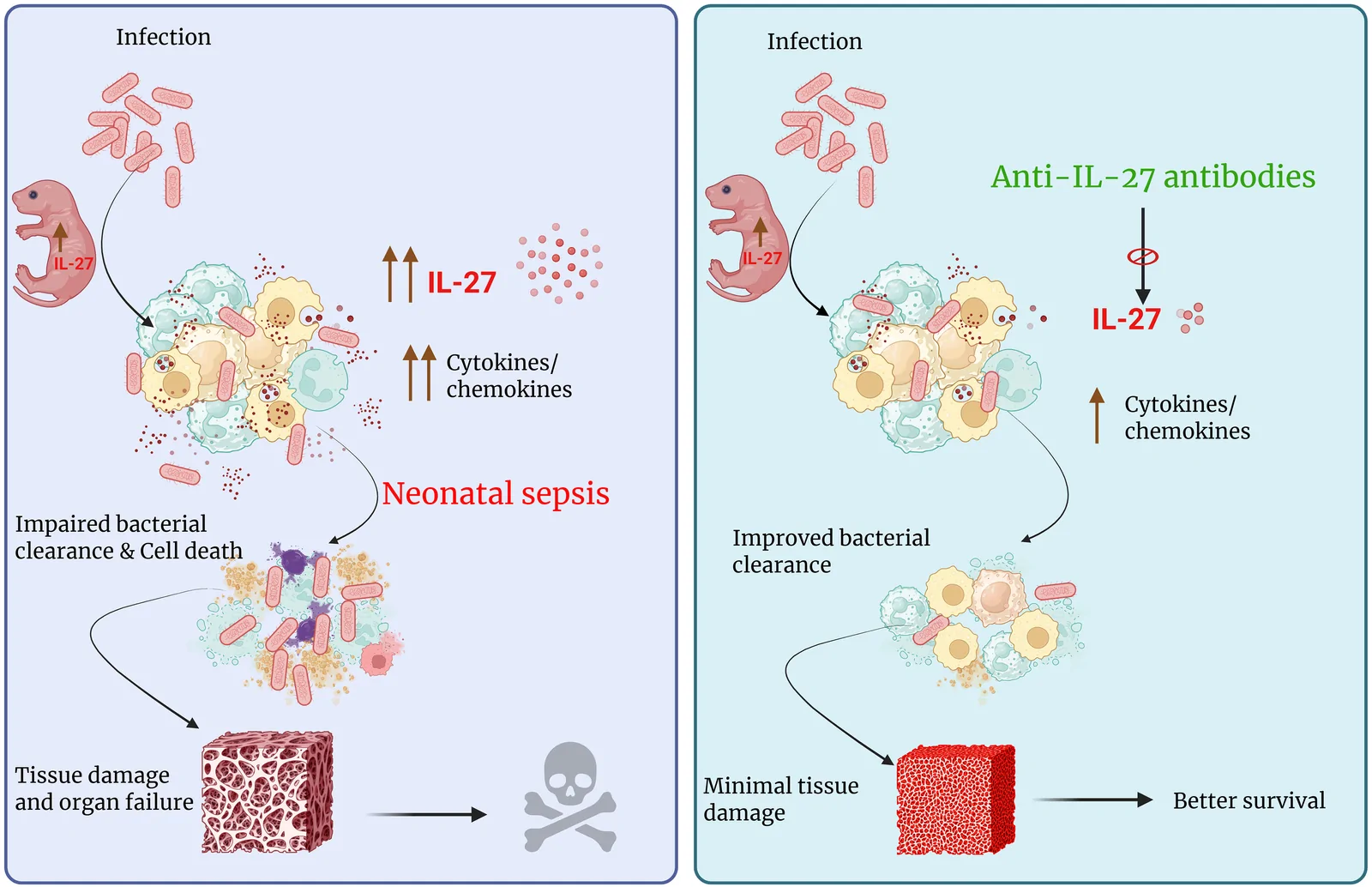

## Introduction

In human development, extensive physiological adaptation occurs during the neonatal period defined by the first 4 weeks of life (1). Among these adaptations, neonates have a developing immune system that is unique from that of adults and is associated with increased susceptibility to infections (2). The WHO reports that infections result in greater than 550,000 neonatal deaths each year (3). Among the most severe infectious conditions in neonates is sepsis, characterized by a blood infection with excessive inflammation and tissue pathology that occurs in an infant younger than 90 days old (4). Neonatal sepsis remains a major challenge globally, with the latest estimates suggesting up to 5 million cases and about 800,000 deaths each year (5); as such, it results in significant mortality and neurodevelopmental impairment among survivors (6). GBS remains the most isolated pathogen in term infants, whereas *E*. *coli* is more commonly responsible for mortality with preterm and low-birth-weight infants during early onset of sepsis (EOS) (7, 8).

Antibiotic therapy and general supportive care are the primary treatment options for neonatal sepsis (9). Common empirical antibiotic regimes with substantial geographical variations include the combinations of a penicillin and an aminoglycoside for EOS and a β-lactam antibiotic or a glycopeptide antibiotic plus an aminoglycoside for late onset of sepsis (10). Emergence of resistant organisms, antibiotic-induced microbiome alterations, and downstream effects of high-dose antibiotics on the developing immune system that lead to short- and long-term health outcomes in neonates are unresolved challenges in the treatment of neonatal sepsis. In particular, necrotizing enterocolitis (NEC) is a serious disease in preterm infants that is linked with antibiotics used in the early weeks of life. These drawbacks necessitate the discovery of new therapeutic strategies and, particularly, those that reduce the dose or longevity of antibiotics (11). Alternative biological agents, such as bacteriophages, antibodies, antivirulence agents, immune-modulating agents, and probiotics, are increasingly being explored as nontraditional alternatives to antibiotics (12).

Cytokines help to coordinate and resolve inflammation during sepsis (13). Though cytokine signaling can stimulate beneficial inflammatory mechanisms designed to clear microbial pathogens, excessive production of proinflammatory cytokines causes an uncontrolled inflammatory response commonly referred to as a “cytokine storm” that leads to tissue damage, multiorgan failure, and death during sepsis (14). Therapies targeting specific cytokines or cytokine pathways have been investigated as potential treatments for sepsis (15–17).

IL-27 is a heterodimeric cytokine that belongs to the IL-6/12 cytokine family (18). IL-27 elicits proinflammatory activity by promoting T cell differentiation to the Th1 lineage and inhibits the differentiation of Th2 cells (19, 20). It also suppresses Th17 cell differentiation and induces the production of the antiinflammatory cytokine IL-10 (21). Reduced inflammatory cytokine production has been reported broadly for myeloid and lymphocyte subsets in response to IL-27 under a variety of acute and chronic stimuli (22–26). Thus, because of its diverse influences on the immune response, researchers have explored the therapeutic efficacy of IL-27 in various diseases, such as cancer and inflammatory diseases (27–30). We and others have reported that IL-27 contributes to enhanced susceptibility to bacterial infection, and inflammatory cytokine responses to Gram-negative bacterial products are increased in neonates (31, 32). In our established neonatal murine sepsis model, we found that IL-27 signaling resulted in increased sepsis-associated morbidity and mortality compared with IL-27 receptor α–deficient neonatal mice that exhibited greater bacterial clearance with reduced systemic inflammation and improved glucose homeostasis (32, 33). Collectively, these findings are supported by evidence in a prospective study of human neonates suspected of infection in which IL-27 levels correlated with early-onset neonatal sepsis disease prediction (34). In the work presented here, we have performed a preclinical study that investigated the efficacy of IL-27 antagonization in rescuing *E*. *coli*–induced neonatal sepsis in mice.

## Results

### In neonatal mice, IL-27p28 antibody treatment results in improved outcomes during E. coli–induced sepsis.

**Figure 1:**
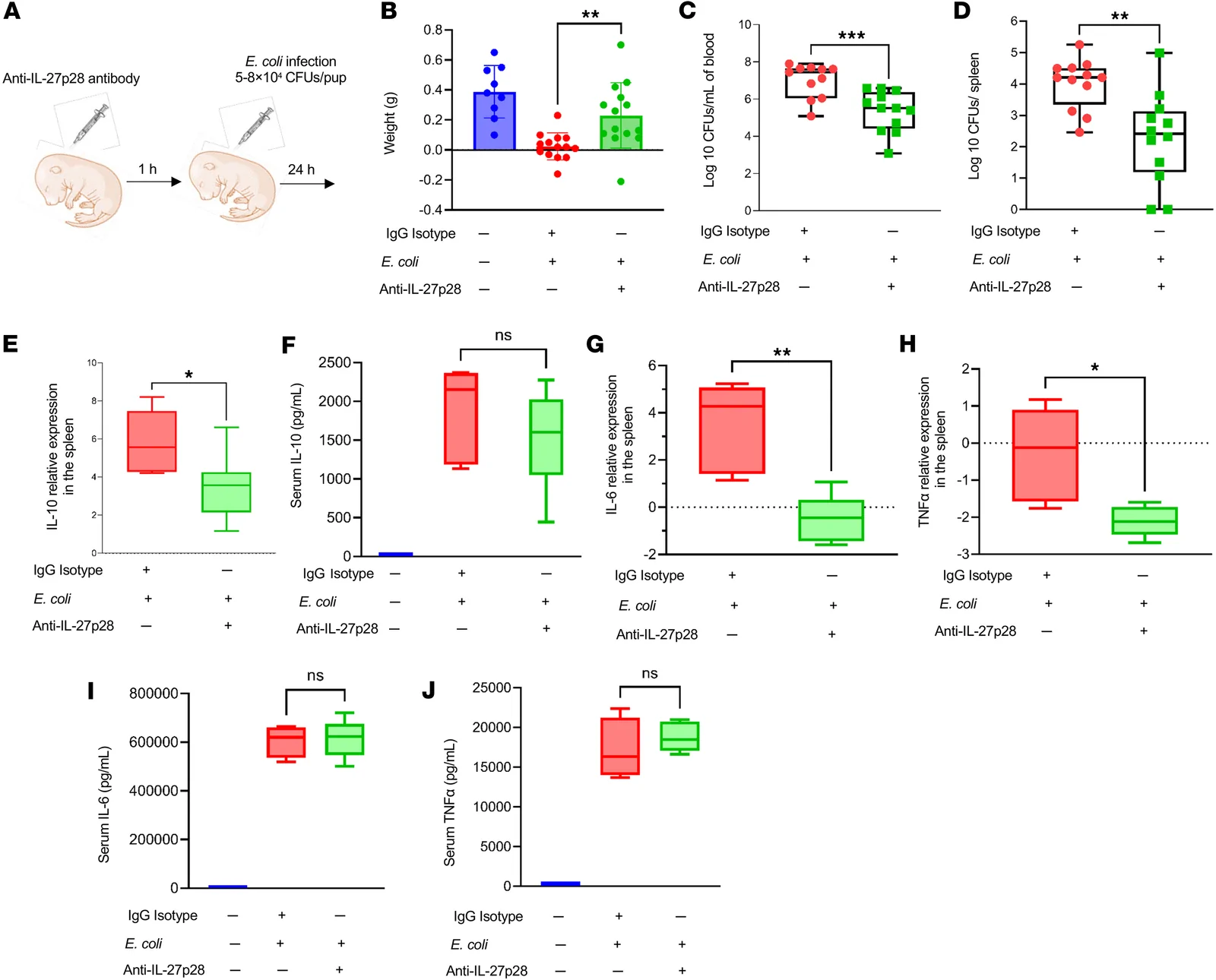
IL-27 neutralization improves outcomes during murine neonatal sepsis induced by *E*. *coli*. (**A**) Neonatal 4-day-old C57BL/6 pups were subcutaneously administered 10 μg anti–IL-27 antibody or isotype control 1 hour prior to infection with an inoculum range of 5 × 10^4^ to 8 × 10^4^ CFUs of *E*. *coli* O1:K1:H7. Body weights were recorded, blood was collected, and spleens were harvested at 24 hours after infection. (**B**) Mean weight ± SD of each experimental group at 24 hours after infection. Median bacterial burdens in the (**C**) blood and (**D**) spleen are shown (*n* = 2–3 pups in each group per experiment across 4 experiments with separate litters, total *n* = 33). (**E** and **F**) Box plots represent the gene expression levels of IL-10 in the spleens and protein levels in the serum of treatment groups. The expression was determined relative to uninfected control spleens by real-time PCR using the formula 2^–ΔΔCt^ (*n* = 2–3 in each group per experiment across 3 experiments with separate litters were used, total *n* = 20). (**G** and **H**) Box plot showing average gene expression of IL-6 and TNF-α in the spleens of treatment groups relative to controls, respectively. Median serum levels of (**I**) IL-6 and (**J**) TNF-α ± SEM measured by multiplex immunoassay are shown for 5 animals in each group from 2 different experiments. Boxes represent the median (center line) and extend from the 25th to 75th percentiles (bottom and top lines, respectively); whiskers extend from the minimum to the maximum value. Statistical significance was determined using ANOVA with Tukey’s multiple comparison (**B**, **F**, **I**, and **J**), Welch’s *t* test (**C**, **D**, **E**, **G**, and **H**). ns, *P* = 0.123; *P = 0.033; **P = 0.002; ***P = 0.0002; ****P = 0.0001.

To evaluate the potential for IL-27 antagonization to improve outcomes in an experimental model of neonatal sepsis, we first investigated administration of antibodies alone as a monotherapy. We initially infected day 4 neonatal mice with an infectious dose of 5 × 10^4^ to 8 × 10^4^ CFUs/pup. Anti–IL-27p28 antibody was administered 1 hour prior to the *E*. *coli* infection (Figure 1A). At 24 hours after infection there was significant weight gain observed in the infected pups that received anti–IL-27p28 compared with uninfected pups (Figure 1B). Infected pups that received anti–IL-27p28 were able to clear bacteria more efficiently from the blood and spleen (Figure 1, C and D). Infected pups had high bacterial burdens in the blood and spleen at 24 hours after infection that were significantly reduced in pups pretreated with IL-27p28 antibody. It is known that IL-27 induces IL-10 production from a wide range of cell types, including T cell subtypes and macrophages (35, 36). In our model of neonatal sepsis induced by *E*. *coli*, we observed elevated gene expression levels of IL-10 in the spleens of infected pups (Figure 1E) (33). Neutralization of IL-27 significantly reduced the gene expression of IL-10 in spleens at 24 hours after infection relative to that in infected pups (Figure 1E); however, in the serum, IL-10 levels were reduced compared with those in infected pups but did not achieve statistical significance (Figure 1F). At 24 hours after infection, pups that received IL-27 antibody showed significantly decreased gene expression levels of cytokines IL-6 and *TNFA* in the spleen compared with infected neonates that did not receive treatment (Figure 1, G–J), although IL-6 and TNF-α protein levels were not different systemically in the serum between treatment groups (Figure 1, I and J).

### IL-27 neutralization enhanced the lysosomal acidification and bacterial clearance of neonatal bone marrow–derived macrophages.

**Figure 2:**
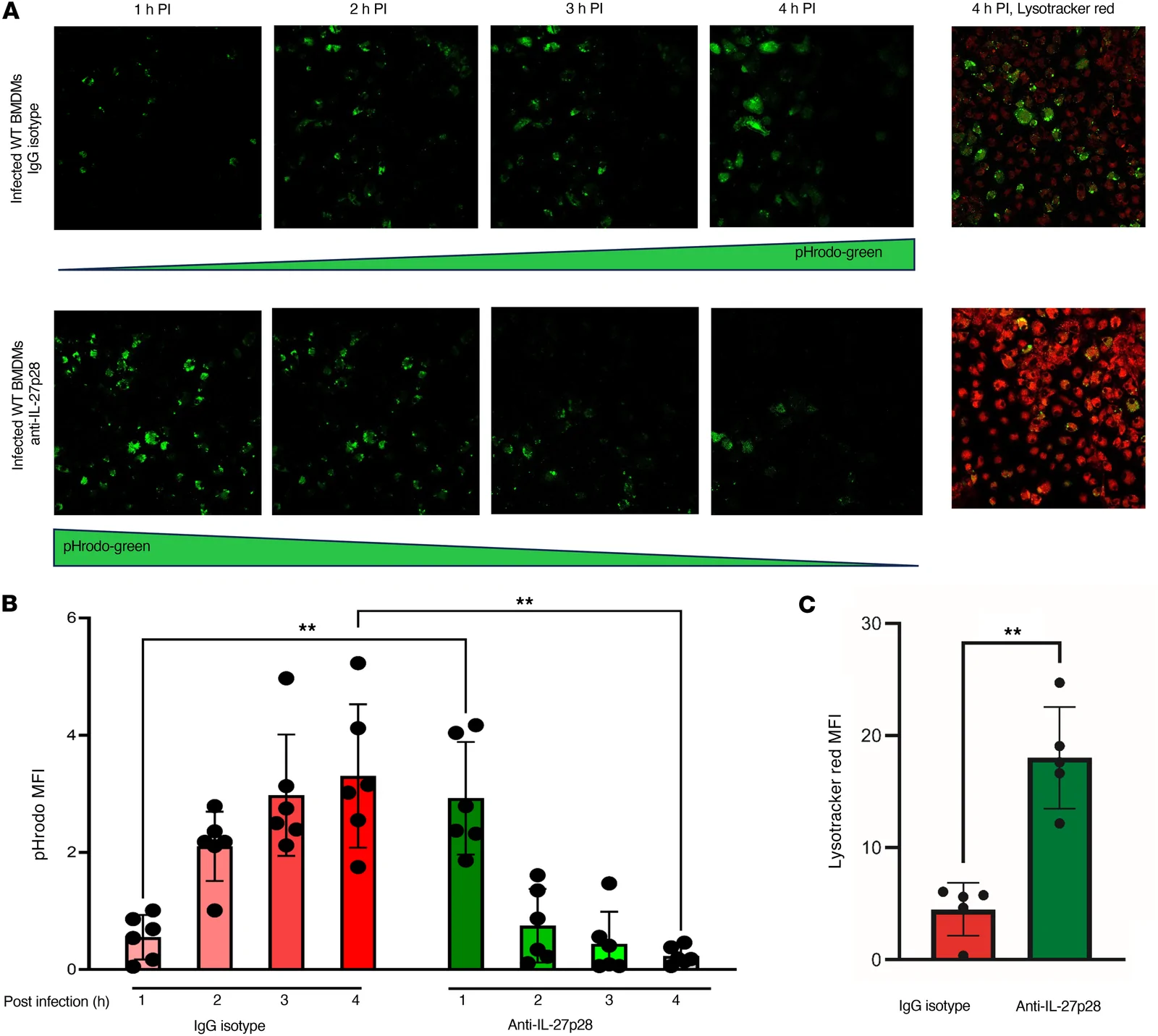
Improved bacterial phagocytosis and trafficking to lysosomes in neonatal BMDMs during IL-27 neutralization. BMDMs from neonatal mice were pretreated with 10 μg/mL anti–IL-27 antibody or isotype control for 16 hours and infected with pHrodo-labeled *E*. *coli* O1:K1:H7 at a multiplicity of infection (MOI) of 25. At 4 hours after infection, LysoTracker Red was added to the culture. (**A**) Representative confocal images (original magnification, ×40) of neonatal BMDMs showing green, fluorescent bacteria inside lysosomes from 1–4 hours after infection. Red fluorescence labels acidified lysosomes. Quantification of green (**B**) and red (**C**) mean fluorescence using ImageJ software from 3 different fields of view from 2 independent experiments (see Supplemental material, section 1). Statistical significance was determined using 2-way ANOVA with Šídák’s multiple comparisons for **B** and unpaired Welch’s *t* test for **C**. **P = 0.002.

IL-27 inhibits the phagolysosomal pathway by reducing the recruitment of vacuolar H+-ATPase (V-ATPase) to lysosomes. This limits lysosomal acidification and impairs the bacterial clearance mechanism of phagocytes in the host (37). To determine the cell-intrinsic mechanism by which IL-27 neutralization improves host defense, we evaluated lysosomal acidification in neonatal macrophages. Within 1 hour after infection, IL-27–neutralized bone marrow–derived macrophages (BMDMs) were able to internalize a significantly greater number of bacteria compared with those that received control antibody (Figure 2, A and B). At 4 hours after infection most of the bacteria were cleared in BMDMs in the absence of IL-27 signaling; in contrast, BMDMs that received control antibody were still increasing the number of bacteria in acidified compartments (as shown in green in Figure 2A), reflecting a delay in bacterial control (Figure 2, A and B). The presence of acidified lysosomes in the live cells was confirmed by lysotracker dye intensity that was significantly increased in the IL-27–neutralized BMDMs (Figure 2C). Overall, the addition of anti–IL-27p28–neutralizing antibody significantly enhanced rapid trafficking of *E*. *coli* to acidified lysosomes.

### Gentamicin treatment rescued septic pups that exhibited similar morbidity and mortality to neonatal mice with genetically disrupted IL-27 signaling.

**Figure 3:**
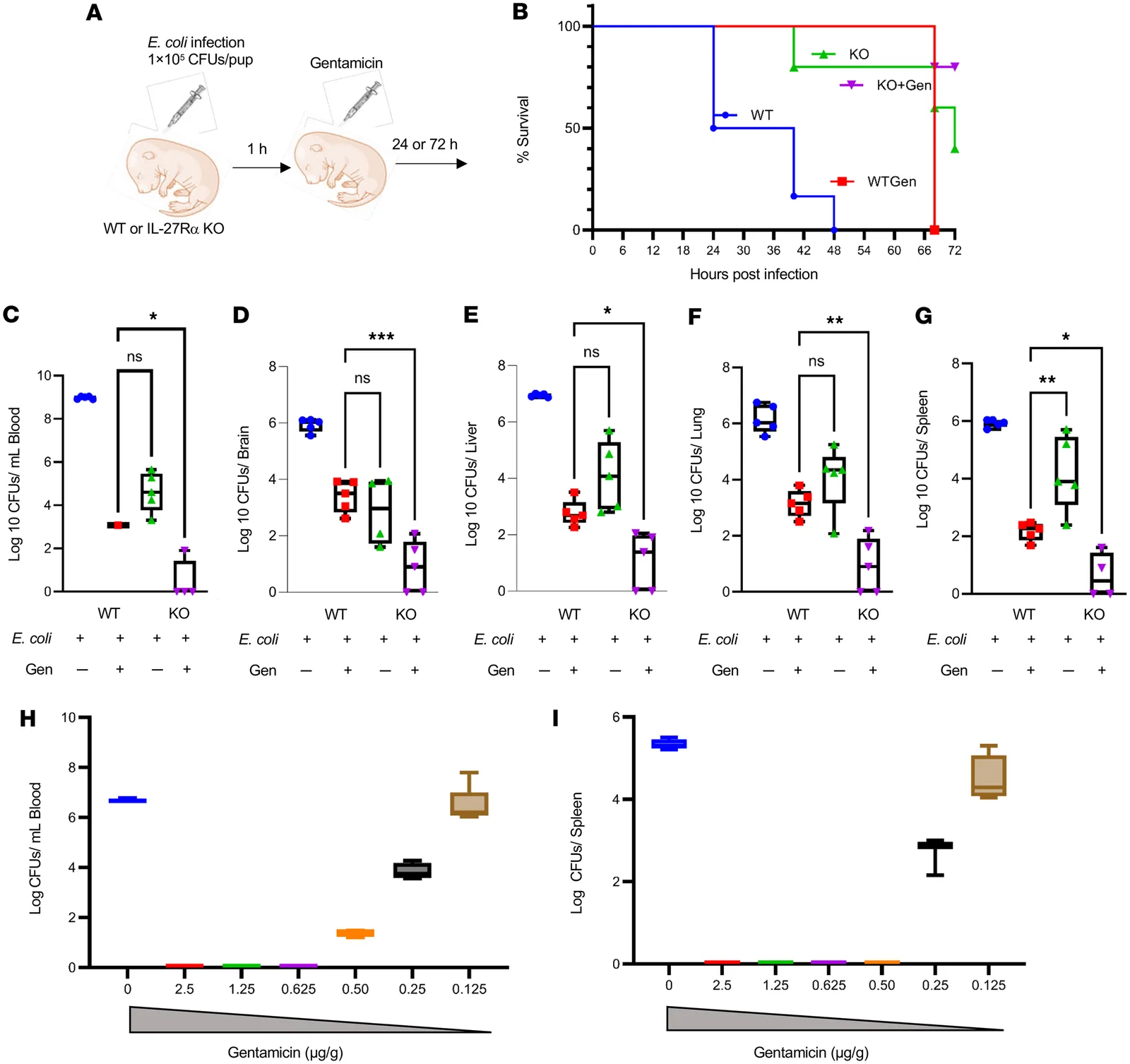
Administration of gentamicin rescues septic pups that exhibit similar morbidity and mortality to neonatal mice that fail to respond to IL-27 signaling. (**A**) Neonatal 4-day-old C57BL/6 (WT) and *Il27ra^–/–^* (KO) pups (*n* = 2–3 per experiment) were infected subcutaneously with a range of 1 × 10^5^ to 2 × 10^5^ CFUs of *E*. *coli* O1:K1:H7. After 1 hour of infection, C57BL/6 pups received 5 μg/g gentamicin (Gen) subcutaneously. (**B**) A Kaplan-Meier survival curve for WT or KO with and without gentamicin over 120 hours of infection (total *n* = 20 across 3 experiments with separate litters). The median (**C**) blood and (**D**–**G**) peripheral organ CFUs are shown (total *n* = 20 across 3 litters with separate litters). For determining the subclinical effective dose of gentamicin, infected neonatal pups were rescued with 2-fold diluted concentrations of gentamicin ranging from 2.5 to 0.125 μg/g. Median blood (**H**) and spleen (**I**) bacterial burdens from a representative experiment are shown. Boxes represent the median (center line) and extend from the 25th to 75th percentiles (bottom and top lines, respectively); whiskers extend from the minimum to the maximum value. Statistical significance was determined using ordinary 1-way ANOVA with Tukey’s multiple comparisons (**C**–**G**). *P = 0.033; **P = 0.002; ***P = 0.0002.

A preferred empiric regimen for early-onset neonatal sepsis includes ampicillin and an aminoglycoside such as gentamicin (38, 39). Recommended doses of gentamicin range from 5 to 7.5 mg/kg (40). Before augmenting antibody neutralization with antibiotic treatment, we first evaluated the efficiency of genetic deletion of IL-27 signaling in controlling infection compared with antibiotic treatment. Both IL-27Ra–KO and WT pups were infected with *E*. *coli*, and after 1 hour, pups were rescued with or without a single dose of gentamicin (5 mg/kg) (Figure 3A). The median survival rate for infected WT pups was 32 hours, which was substantially increased with antibiotic treatment to 68 hours (Figure 3B). Comparatively, infected KO pups had a median survival rate of 72 hours without antibiotic treatment (Figure 3B) and 80% survival rate with antibiotic treatment. Furthermore, KO pups were as efficient as gentamicin-treated WT pups in clearing bacteria from the blood and peripheral tissues (Figure 3, C–G). Complete clearance of bacteria was observed in 40% of the KO pups that received gentamicin (Figure 3, C–G).

To observe the potential added benefit of IL-27 neutralization, we needed to determine suboptimal rescue concentrations of gentamicin in our *E*. *coli*–induced neonatal sepsis model. We administered serially diluted concentrations of gentamicin to neonatal pups 1 hour after infection. The last concentration to provide full protection with CFUs in the blood and spleen at or below the limit of detection was 0.625 mg/g (0.625 mg/kg) (Figure 3, H and I). Infected pups that received 0.5 and 0.25 mg/g gentamicin showed detectable bacterial burdens in the blood and/or spleen, albeit reduced from infected pups in the absence of treatment, with moderate signs of sickness compared with infected pups (Figure 3, H and I). A lower concentration of 0.125 μg/g (0.125 mg/kg) body weight of gentamicin at 1 hour after infection left infected pups unable to clear the blood and tissue bacterial burdens (Figure 3, H and I) at 24 hours after infection. These pups also exhibited signs of morbidity such as dehydration, poor feeding, and lethargy.

### IL-27p28 antibody complements gentamicin to promote efficient bacterial clearance with improved morbidity and reduced cytokine levels in septic pups.

**Figure 4:**
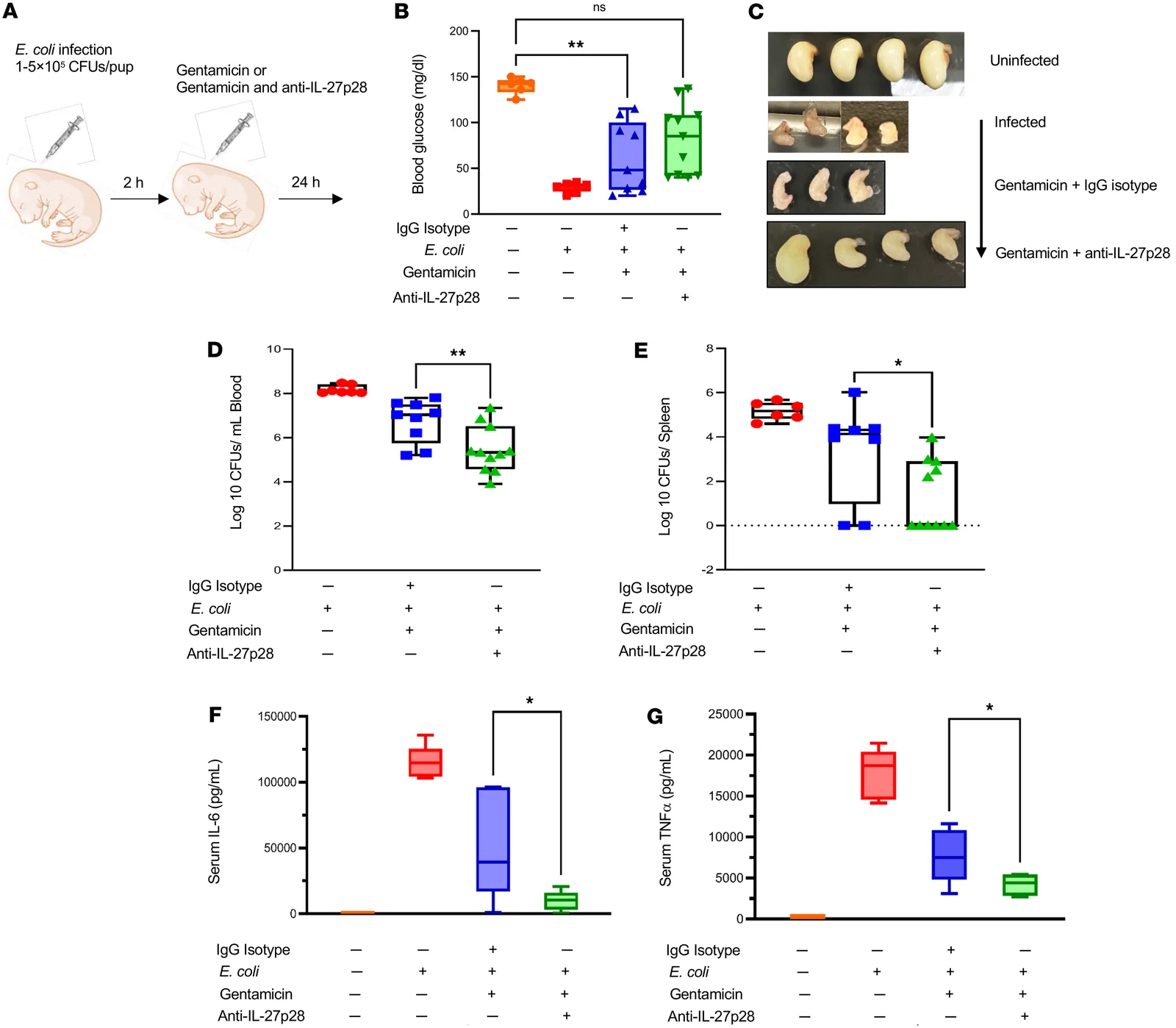
IL-27 neutralization augments gentamicin to improve bacterial clearance, neonatal morbidity, and reduced systemic inflammation. (**A**) Neonatal 4-day-old C57BL/6 mice pups (*n* = 2–3 per experiment) were infected subcutaneously with a range of 1 × 10^5^ to 5 × 10^5^ CFUs of *E*. *coli* O1:K1:H7. After 2 hours of infection, pups received no treatment, 5 μg/g gentamicin alone, or a combination with 10 μg IL-27 monoclonal antibody subcutaneously. Blood and peripheral organs were collected at 24 hours after infection. (**B**) Box plot with blood glucose levels for each pup is shown. (**C**) The stomachs of pups that received IL-27 antibody demonstrated reduced gross pathology and increased milk availability. The median (**D**) blood and (**E**) spleen CFUs are shown (total *n* = 32 across 4 experiments with separate litters). (**F** and **G**) Box plot showing average serum levels of IL-6 and TNF-α in the spleens of treatment groups, respectively, measured by multiplex immunoassay for each pup (total *n* = 20 across 3 experiments with separate litters). For all the box plots, boxes represent the median (center line) and extend from the 25th to 75th percentiles (bottom and top lines, respectively); whiskers extend from the minimum to the maximum value. Statistical significance was determined using Kruskal-Wallis’s test with Dunn’s multiple comparisons (**B**), ordinary 1-way ANOVA with Tukey’s multiple comparisons (**D** and **E**), and ordinary 1-way ANOVA with Holm-Šídák multiple comparisons (**F** and **G**). ns, *P* = 0.123; *P = 0.033; **P = 0.002.

To evaluate the efficacy of antibody combined with antibiotic, pups were challenged with a high infectious dose of *E*. *coli*, 1 × 10^5^ to 5 × 10^5^ CFUs/pup (Figure 4A). At 24 hours after infection, pups were severely morbid with a lack of mobility, hypoglycemia, and no milk spot visible as evidence of limited hydration and ability to nurse (Figure 4, B and C). Pups that received a subclinical concentration of gentamicin (0.5 mg/g) with control antibody at 2–4 hours after infection displayed significantly reduced glucose levels compared with uninfected pups, though they were not as morbid as infected pups that did not receive antibiotic (Figure 4, B–E). Pups that were given the combination of gentamicin and IL-27p28 antibody were able to maintain blood glucose levels, and active feeding was evidenced by a prominent milk spot and full stomach that was absent in the group treated with antibiotic alone (Figure 4, B and C). Bacterial proliferation in the blood and spleen was significantly restricted with the IL-27–targeted combination treatment relative to that of pups that received antibiotic with control antibody (Figure 4, D and E). Serum IL-6 and TNF-α levels were significantly low in gentamicin and IL-27p28 antibody–treated pups during infection compared with the group treated with gentamicin alone (Figure 4, F and G).

### IL-27p28–neutralizing antibody improved the efficacy of low-dose antibiotic treatment to confer protection from septic lethality and prevent tissue pathology.

**Figure 5:**
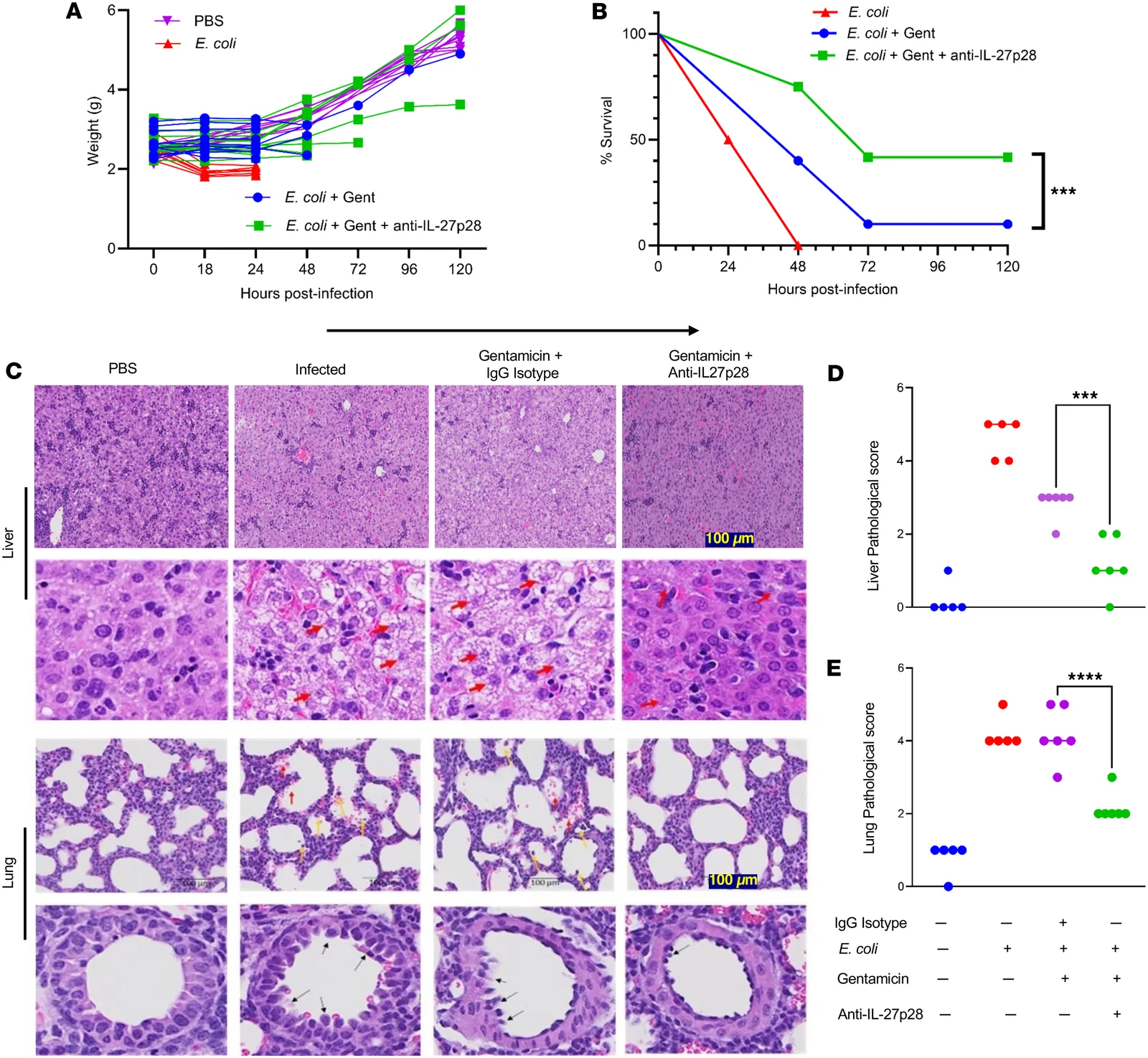
Improved survival with minimal liver and lung damage in septic pups that received the combination of gentamicin and IL-27 antibody. Neonatal 4-day-old C57BL/6 mice pups (*n* = 2–3 per experiment) were infected subcutaneously with a range of 1 × 10^5^ to 5 × 10^5^ CFUs of *E*. *coli* O1:K1:H7. After 2 hours of infection pups received no treatment, 0.5 μg/g gentamicin alone, or a combination with 10 μg IL-27 monoclonal antibody subcutaneously. Pups were weighed and monitored for morbidity and mortality through 120 hours of infection. (**A**) The weight is shown for the time survived for each pup. (**B**) The percentage survival of each experimental group over time (total *n* = 37 across 5 experiments with separate litters). Statistical significance was determined using simple survival analysis (Kaplan Meier) with log rank (Mantel-cox) test. ***P = 0.0003. Livers and lungs were harvested at 24 hours after infection for H&E staining and histopathology (total *n* = 22 across 3 experiments with separate litters). (**C**) Representative liver and lung tissue sections from each experimental group as indicated are shown. Enlarged regions of the same liver sections are shown, with red arrows indicating necrotic and degrading cells. Red arrows in the lung sections indicate alveolar hemorrhaging, and yellow arrows point to desquamation of alveolar epithelial cells into the alveolar spaces in the enlarged regions of lung sections. The same lung sections show that highlight hypertrophic (black arrows) airway epithelial cells. Scale bar: 100 μm. Compiled liver (**D**) and lung (**E**) histology scoring are shown. Statistical significance was determined using ordinary 1-way ANOVA with Tukey’s multiple comparisons. ***P = 0.0002; ****P = 0.0001.

To evaluate if more efficient bacterial clearance and other improved outcomes during neonatal sepsis at 24 hours in response to IL-27p28 antibody and subclinical antibiotic therapy enhanced survival, we monitored pups through 120 hours of infection. Infected pups without antibiotic struggled to maintain the body weight in the first day, and all weighed less than the start of infection (Figure 5A). Half of these animals succumbed to infection within the first day, and the remaining animals succumbed by 48 hours after infection (Figure 5B). Administration of 0.5 mg/g gentamicin combined with control antibody promoted maintenance of body weight through 24 hours, but 80% of the pups reduced weight thereafter (Figure 5A). Conversely, 75% of the pups were able to gain weight throughout infection when given IL-27p28–neutralizing antibody and gentamicin (Figure 5A). Supplementing gentamicin with IL-27p28 antibody significantly increased the 5-day survival rate from 10% to 41.6% (Figure 5B).

In conjunction with the high mortality rate, severe sepsis is also associated with tissue damage and organ failure (41). Histopathological analysis of H&E-stained liver sections from infected pups with and without gentamicin alone presented with necrotic regions and increased cell death (Figure 5, C and D). In the lungs, alveolar hemorrhage along with desquamation of epithelial cells into the alveolar spaces was prominent (Figure 5, D and E). In contrast, treatment with IL-27p28 neutralization combined with gentamicin maintained liver and lung tissue architecture with minimal damage (Figure 5, C–E).

## Discussion

Sepsis is responsible for significant morbidity and mortality in the neonatal population, and therapeutic options for these infections are limited (42). Antibiotic therapy remains the only first-line treatment arsenal beyond supportive care for neonatal sepsis, while major concerns are associated with evolving multidrug-resistant pathogens. In addition, long-term usage of antibiotics in neonates can cause NEC, a serious gastrointestinal condition (43). Recent advances in development of new neonatal sepsis treatments emphasize immunomodulatory approaches (44). Intravenous immunoglobulin and granulocyte colony-stimulating factor are being investigated as potential adjuvant therapies (45). An important consideration is that neonates have distinct immune profiles and functions compared with adult immunity (46). Untrained immune cells with lower antimicrobial potential increase neonatal susceptibility to infection (47). With the aim to modulate the immature immune response of neonates to combat bacterial infections more efficiently, this study focused on the potential of IL-27 neutralization at the onset of neonatal infection.

We have established in previous studies using neonatal murine models that IL-27 compromises host control of bacteria consistent with elevated levels of inflammatory cytokines and increased mortality during sepsis (32, 33, 48). Conversely, mice that lack IL-27 receptor α, and cannot respond to the cytokine, exhibited improved pathogen clearance in peripheral tissue, reduced systemic inflammation, and limited mortality in septic neonatal mice (33). Using the same KO mouse model another scientific group observed improved bacterial clearance compared with WT that of controls during *S*. *aureus* infection, albeit in adult animals (49). The present investigation evaluated the therapeutic efficacy of IL-27p28 antibody to neutralize elevated levels of IL-27 cytokine present in septic neonatal animals. Consistent with our previous findings in mice that lack the ability to respond to IL-27, prophylactic administration of IL-27p28 antibody to neonatal mice significantly improved bacterial clearance during *E*. *coli*–induced sepsis. IL-27 signaling impairs bacterial clearance through multiple mechanisms. One such important mechanism is the negative regulation of the expression of V-ATPases, which are recruited to late endosomes and lysosomes to promote acidification that is required to eliminate bacteria internalized through phagocytosis (50). Consistent with our findings, Bosmann and colleagues found that blocking of IL-27p28 during polymicrobial sepsis induced by cecal ligation and puncture resulted in improved bacterial clearance in blood and the peritoneal compartment along with improved survival rates and restricted cytokine release in adult mice (51). However, this work was done in adult animals. The impact of IL-27 blockade with soluble neutralizing reagents in neonates had not previously been explored.

Our findings provide a mechanistic framework for neonatal sepsis that IL-27 acts as a molecular gatekeeper that prevents the maturation of the phagolysosome. In the developmentally immature neonatal environment, high levels of IL-27p28 inhibit the V-ATPase–mediated acidification of lysosomes. Neutralizing antibody effectively activated the neonatal macrophage, allowing it to transition from a tolerant state to a bactericidal state. This restoration of killing capacity rather than a purely systemic or metabolic shift is the primary driver of the superior bacterial clearance and survival observed in our combination therapy.

The cytokine response is critical for establishing the severity and outcome of sepsis in newborns (52). In neonates, cytokines such as IL-6, TNF-α, and IL-1β are rapidly and significantly increased upon sepsis onset and characterized by a dysregulated inflammatory response that causes systemic inflammatory response syndrome that can contribute to organ damage (52, 53). A regulated cytokine response is essential for effective clearance of bacteria whereas overproduction of proinflammatory cytokines can lead to impaired function of immune cells (54). Previous studies from our group showed that IL-27 indirectly promotes an inflammatory cytokine response during neonatal sepsis, likely through impaired clearance of bacteria and microbial products. Accordingly, mice deficient in IL-27 signaling exhibited improved bacterial clearance and reduced levels of inflammatory cytokines (32, 33, 48, 55). In this study, pups that received IL-27p28 antibody displayed regulated and balanced proinflammatory and antiinflammatory cytokine expression levels in the spleen during infection consistent with improved bacterial clearance. A study of bacterial pneumonia caused by *Streptococcus pneumoniae* demonstrated that blocking signaling of IL-20 cytokine through its receptor improved bacterial clearance and decreased inflammatory reactions and tissue lesions (56). At the local tissue level there was a significant decrease in inflammatory cytokine gene expression in the spleen with IL-27 neutralization alone at 24 hours after infection in pups that received a moderate infectious dose. We did not observe significant changes in IL-6, IL-10, and TNF-a serum cytokine levels. Though significant bacterial clearance was observed with IL-27p28–neutralizing antibody treatment in the blood compared with infected pups that received control antibody, a single dose of IL-27p28–neutralizing antibody prophylactically was not sufficient to clear bacteria completely from the blood.

Experimental treatment failures and barriers to novel therapeutic approaches have left antibiotic therapy as the sole first-line option to treat infections (57). This study explored a method of supporting antibiotic treatment with an immune modulator for better prognosis of sepsis-related outcomes in neonates. Neonates are highly sensitive to medication, and use of higher doses or longer duration of antibiotics in neonates causes organ-specific damage (ototoxicity, nephrotoxicity, and NEC) and long-lasting dysbiosis and impairs immune development. Additionally, a study found that higher antibiotic exposure in early life has been associated with an increased risk for several chronic conditions, such as respiratory allergies, atopic dermatitis, inflammatory bowel disease, and attention deficit hyperactivity disorder (58, 59). IL-27 neutralization improved clinical outcomes of murine neonatal sepsis with lower doses of antibiotic and, as such, may represent a regimen that reduces the risk of NEC and other consequences of heavy antibiotics early in life. While our study did not directly assess intestinal pathology or gut barrier integrity following gentamicin treatment, the potential risks of antibiotic administration in this model justify critical discussion. Aminoglycosides like gentamicin are effective against Gram-negative septicemia, yet they are known to cause profound dysbiosis by depleting protective commensal microbiota. Given the documented risks of aminoglycoside-induced gut toxicity and the association of early empiric antibiotics with NEC in clinical settings, strategies to minimize antibiotic exposure are heavily warranted. Our results demonstrated that IL-27p28 antibody treatment improved the efficacy of a subclinical dose of gentamicin and increased the survival rate of the neonatal septic mice. Pups that received the combination of gentamicin and IL-27p28 antibody had significantly improved bacterial clearance with a reduced inflammatory response, limited tissue damage, and improved outcomes during sepsis compared with pups that received antibiotic alone. Though we did not detect significant changes in the serum cytokine levels with IL-27 neutralization alone in the infected pups at 24 hours after infection, the combination of antibiotic and antibody was effective in significantly decreasing levels of serum proinflammatory cytokines IL-6 and TNF-α. Several studies have used antibodies designed to target specific bacterial toxins or virulence factors, enhancing the effectiveness of antibiotic treatment with consideration for the risk of antimicrobial resistance (60). However, our study focused on improving host response by suppressing a cytokine that increases susceptibility and opposes bacterial clearance during sepsis in neonates. Other researchers have explored the diagnostic accuracy of IL-27 and proposed the cytokine as a promising biomarker for neonatal sepsis (34, 61). The work presented here has extended those findings to validate IL-27 as a therapeutic target.

This combination therapy could effectively treat severe sepsis while minimizing collateral damage to the developing gut microbiome and intestinal barrier.

Limitations of the study include low the number of observations in each experimental group, which is a common obstacle with neonatal models in which a maximum 8–9 pups per litter limits 2–3 observations for each experimental group. This study used subcutaneous administration of either antibiotic or antibody, as intravenous or intraperitoneal administration on 4-day-old pups is technically challenging and involves either backflow of the injecting material or increased risk of death in the pups. One of our future directions includes standardizing intravenous administration of neutralizing antibody to achieve maximum efficacy. This study has evaluated the efficacy of IL-27 neutralization in combination with one antibiotic, gentamicin, using *E*. *coli* strain O1:K1:H7. This study can also be extended with different antibiotic choices (ampicillin, cefotaxime, vancomycin, etc.) used to treat early onset of neonatal sepsis using other bacterial pathogens, including clinical isolates. In this study, we utilized a monoclonal antibody targeting the IL-27p28 subunit. It is important to note that, because the p28 chain functions both independently as IL-30 and in tandem with EBI3 to form IL-27, our therapeutic intervention may have resulted in the simultaneous neutralization of both cytokine pathways. While IL-30 has been reported to alleviate experimental sepsis by inducing protective IL-10 production in NKT cells (30), the clinical effect of neutralizing p28 generally yields a net survival benefit in our neonatal sepsis model. Although the levels of IL-30 or ratio to IL-27 in our model are unknown, our findings would suggest that the immunosuppressive burden typically driven by the IL-27 heterodimer overrides the protective, antiinflammatory influences of independent IL-30 during active hyperinflammatory septic conditions. Our strategy of applying a functional IL-27p28 blockade directly mirrors phenotypic observations established in genetic Il27ra^–/–^ models, which inherently cannot differentiate between the loss of standalone IL-30 and the active IL-27 heterodimer. This makes our pharmacological intervention directly comparable to established KO literature. To fully explain the isolated therapeutic potential of removing IL-27 while preserving IL-30 in this neonatal sepsis model, future studies should employ EBI3-specific antibodies or IL-27 heterodimer-specific neutralizing clones.

### Conclusions.

Overall, our data suggest that the blockade of IL-27 signaling is beneficial to combat *E*. *coli*–induced sepsis in neonatal mice and can be augmented with antibiotic treatment. This strategy may have additional value with drug-resistant bacteria and other clinical isolates associated with neonatal sepsis.

## Methods

### Sex as a biological variable.

Our study examined male and female 4-day-old pups for experimental infection, and similar findings are reported for both sexes.

### Mice.

C57BL/6 (WT) and Il27ra^–/–^ (KO) mice were purchased from The Jackson Laboratory and maintained under specific pathogen–free conditions in the vivarium at West Virginia University Health Sciences Center. Mice were maintained on a 12-hour-light/dark cycle and were fed and given access to water ad libitum.

### Neonatal murine sepsis infection model.

*E*. *coli* strain O1:K1:H7 was obtained from the ATCC and grown in Luria broth from a single colony isolated on tryptic soy agar. To prepare infectious inoculums, the bacteria were enumerated as described previously (21). Neonatal pups (*n* = 3 or 4 per group [uninfected control, infected with or without antibiotics with or without anti–IL-27p28, or isotype control] per genotype [WT vs. KO]) at 4 days of age were inoculated subcutaneously in the scapular region with 50 μL of either PBS or *E*. *coli* O1:K1:H7 using a 28-gauge insulin needle as described previously (20, 21). The actual infectious dose for each experiment was determined by standard plate counts. Control and treated pups were identified by tail snips and tattoo markings, respectively. The weights of mice were recorded prior to infection and at 24 hours after infection just before euthanasia. For survival experiments, pups were observed for 5 days after infection, and weights were recorded every 6 hours, accompanied by monitoring for signs of morbidity and mortality. This included poor weight gain, lack of spontaneous movements, absence of milk spot, and dehydration. All downstream experiments were associated with the same preidentified mice. Organs were collected and placed in PBS or TriReagent (Sigma Aldrich) or 10% neutral buffered formalin solution for downstream analysis. Blood was deposited in tubes that contained 5 μL of 500 mM ethylenediamine tetraacetate acid and placed on ice. The bacterial burden in the blood and spleen was enumerated by serial dilution and standard plating on tryptic soy agar. Bacterial counts were transformed to log_10_ CFU/mL to normalize the data and stabilize variance for statistical analysis. Blood glucose levels were measured using an AlphaTrack3 blood glucose monitoring system (Zoetis).

On the basis of previous studies by other research groups and initial standardization in our neonatal model, to neutralize IL-27, 10 μg anti–IL-27p28 monoclonal antibody (516912, Biolegend) was subcutaneously injected into each pup 1 hour prior to infection or 1–4 hours following infection (51). Infected control mice were injected with an equivalent amount of IgG2 mouse isotype control antibody (400264, Biolegend) prior to infection. For experiments in which pups were rescued with antibody alone, a lower infectious dose of 5 to × 10^4^ 8 × 10^4^ CFUs was delivered. For antibiotic administration, a sublethal dose of gentamicin, at which pups were unable to clear the bacterial burden, was determined by administering 2-fold dilutions of gentamicin in the range 2.5–0.125 μg/g at 1 hour after infection. We determined that for the infectious dose of 10^5^ CFUs/pup, the gentamicin concentration of 0.5 and 0.25 μg/g at 1 hour after infection is an ideal sublethal dose. For antibody and gentamicin combination studies, we selected 0.5 μg/g gentamicin concentration at 2 hours after infection. In subsequent experiments, infected pups were rescued by administering 0.5 μg/g gentamicin alone or in combination with 10 μg anti–IL-27p28 antibody at 2 hours after infection. Infection outcomes and treatment efficiencies were assessed 24 hours after infection, and survival studies were conducted for 5 days. For experiments in which pups were treated with gentamicin alone or in combination with antibody, they were challenged with an infectious dose of 1–5 × 10^5^ CFUs/pup.

### Bacterial phagocytosis study.

Neonatal bone marrow isolation and differentiation into macrophages (BMDMs) was performed as described previously (62). BMDMs were seeded in a 35 mm quad dish (iBidi) at a density of 2 × 10^5^ cells per quadrant in a volume of 500 μL complete DMEM. Cells were treated either with 10 μg/mL IgG isotype or anti–IL-27 antibody for 16 hours. The bacterial inoculum was prepared at the multiplicity of infection of 30 using a pretitered stock of *E*. *coli* O1:K1:H7 stored at –80°C. Bacteria were labeled with the 500 μM pH-sensitive dye pHrodo green (Thermo Fisher Scientific) for 20 minutes. The bacterial pellet was resuspended in 500 μL complete DMEM without phenol red and added to the BMDMs along with either IgG isotype or anti–IL-27 antibody. After 1 hour after infection medium was then replaced with fresh medium supplemented with gentamicin (100 μg/mL). Immediately cells were visualized using the Zeiss 710 confocal microscope with 3 fixed positions in each treatment group to observe phagocytosis in the same field of view for 4 hours by incubating at 37°C with 5% CO_2_. Images were acquired each hour. After 4 hours of infection, 200 ng LysoTracker Red (Thermo Fisher Scientific) was added to observe acidified lysosomes.

### RNA isolation and gene expression analysis.

Spleens were thawed and homogenized in TriReagent (Sigma Aldrich). RNA was isolated using the commercial product protocol. Briefly, the upper aqueous layer following phase separation was mixed with an equal volume of 75% ethanol and transferred to E.Z.N.A. RNA isolation columns (Omega Biotek). The manufacturer’s instructions were followed to complete tissue RNA isolation. iScript cDNA synthesis reagents (Bio-Rad) were used to generate first-strand cDNA according to the manufacturer’s protocol. Real-time cycling of reactions that included cDNA from the above preparation diluted 1:3 in nuclease-free water, gene-specific primer probe sets (Applied Biosystems), and iQ Supermix (Bio-Rad) was performed in triplicate using a StepOnePlus (Applied Biosystems) real-time detection system. Cytokine-specific amplification was normalized to that of *actB* as an internal reference gene and expressed as log_2_ relative gene expression compared with control spleens using the formula 2^–ΔΔCt^.

### Cytokine detection.

IL-6 and TNF-α serum levels were measured using multiplexed electrochemiluminescence U-plex reagents according to the manufacturer’s protocol (MesoScale Discovery [MSD]). Results were analyzed using MSD Discovery Workbench software (v4.0.13). Protein standards were assayed in parallel with samples.

### Histopathology.

Lung and liver tissues were post-fixed in 10% neutral buffered formalin, embedded in paraffin, sectioned at a thickness of 3 μm, and then stained with H&E solution according to standard procedures performed by the Electron Microscopy Histopathology and Tissue Bank Core Facility at West Virginia University. The stained slides were covered with coverslips. Images were captured by light microscopy (Olympus slide scanner). Blinded histological scoring of liver and lung was performed by following multiparametric and semiquantitative scoring systems (normal to severe inflammation, 0 –5) (63). (Details are provided in the supplemental material, section 2; supplemental material available online with this article; https://doi org/10.1172/jci.insight.204603DS1).

### Statistics.

Statistical analysis was performed with GraphPad Prism version 8. The sample size for each experiment is specified in the figure legends, and each experiment was repeated a minimum of 3 times. Data are expressed as mean ± SEM, as indicated in the figure legends, and were tested using 1-way ANOVA, (utilizing appropriate follow-up multiple comparisons tests) or 2-tailed Student’s *t* test. The threshold for statistical significance was set at α = 0.05.

### Study approval.

All procedures were approved by the West Virginia University Institutional Animal Care and Use Committee (protocol 1708008935) and conducted in accordance with the recommendations from the *Guide for the Care and Use of Laboratory Animals* by the National Research Council (National Academies Press, 2011).

### Data availability.

No datasets were generated or used in the current study. Underlying data are available in the Supporting Data Values file.

## Author contributions

MA: writing the original draft of the manuscript, manuscript review and editing, investigation, project administration, formal analysis, data curation, validation, methodology, conceptualization, and visualization. JMP: manuscript review and editing and investigation. SAM: histopathology scoring and review. CMR: software, formal analysis, funding acquisition, resources, visualization, supervision, project administration, conceptualization, methodology, and manuscript review and editing.

## Conflict of interest

The authors have declared that no conflict of interest exists.

## Funding support

This work is the result of NIH funding, in whole or in part, and is subject to the NIH Public Access Policy. Through acceptance of this federal funding, the NIH has been given a right to make the work publicly available in PubMed Central.

- NIH grants AI154129 and AI163333 to CMR.

## Supplementary Material

Supplemental data

Supporting data values

## Version 1 07/23/2026

In-Press Preview

## Version 2 09/08/2026

Electronic publication
