# Supplementary material for: IL-27 neutralization with and without antibiotics as an approach to prevent and treat neonatal sepsis
Source: JCI Insight. 2026 Jul 23;11(17):e204603. doi: 10.1172/jci.insight.204603 (PMC13564075; doi:10.1172/jci.insight.204603)
Supplement: Supplementary file 1 [file jciinsight-11-204603-s137.pdf]

1. Lysosomal acidification data

Green MFI measurements

			Green MFI			
1	WT1h1.czi - C=0	125490.76	0.17	6.57	0	255
2	WT1h2.czi - C=0	125490.76	0.54	11.74	0	255
3	WT1h3.czi - C=0	125490.76	0.05	3.7	0	255
4	WT1h1.czi - C=0	125490.76	0.86	14.74	0	255
5	WT1h2.czi - C=0	125490.76	1.01	16.05	0	255
6	WT1h3.czi - C=0	125490.76	0.69	13.25	0	255
7	WT2h1.czi - C=0	125490.76	1.01	15.98	0	255
8	WT2h2.czi - C=0	125490.76	2.79	26.53	0	255
9	WT2h1.czi - C=0	125490.76	2.36	24.4	0	255
10	WT2h2.czi - C=0	125490.76	2.11	23.08	0	255
11	WT2h3.czi - C=0	125490.76	2.18	23.46	0	255
12	WT3h1.czi - C=0	125490.76	2.39	24.56	0	255
13	WT3h2.czi - C=0	125490.76	2.12	23.17	0	255
14	WT3h3.czi - C=0	125490.76	2.75	26.32	0	255
15	WT3h1.czi - C=0	125490.76	4.97	35.25	0	255
16	WT3h2.czi - C=0	125490.76	2.5	25.13	0	255
17	WT3h3.czi - C=0	125490.76	3.13	28.07	0	255
18	WT4h1.czi - C=0	125490.76	1.75	21.02	0	255
19	WT4h2.czi - C=0	125490.76	3.02	27.61	0	255
20	WT4h3.czi - C=0	125490.76	2.55	25.37	0	255
21	WT4h1.czi - C=1	125490.76	5.23	36.13	0	255
22	WT4h2.czi - C=1	125490.76	4.12	32.16	0	255
23	WT4h3.czi - C=1	125490.76	3.15	28.17	0	255
24	WT+Neut1h1.czi	125490.76	1.86	21.7	0	255
25	WT+Neut1h2.czi	125490.76	2.32	24.22	0	255
26	WT+Neut1h3.czi	125490.76	2.79	26.52	0	255
27	WT+Neut1h1.czi	125490.76	2.37	24.47	0	255
28	WT+Neut1h2.czi	125490.76	4.17	32.33	0	255
29	WT+Neut1h3.czi	125490.76	4.04	31.85	0	255
30	WT+Neut2h1.czi	125490.76	0.22	7.47	0	255
31	WT+Neut2h2.czi	125490.76	0.34	9.25	0	255
32	WT+Neut2h3.czi	125490.76	0.11	5.28	0	255
33	WT+Neut2h1.czi	125490.76	0.87	14.91	0	255
34	WT+Neut2h3.czi	125490.76	1.61	20.18	0	255
35	WT+Neut2h2.czi	125490.76	1.35	18.48	0	255
36	WT+Neut3h1.czi	125490.76	0.56	11.94	0	255
37	WT+Neut3h2.czi	125490.76	0.09	4.74	0	255
38	WT+Neut3h3.czi	125490.76	0.06	3.97	0	255
39	WT+Neut3h1.czi	125490.76	0.41	10.17	0	255
40	WT+Neut3h2.czi	125490.76	0.06	3.99	0	255
41	WT+Neut3h3.czi	125490.76	1.47	19.29	0	255
42	WT+Neut4h1.czi	125490.76	0.46	10.86	0	255
43	WT+Neut4h2.czi	125490.76	0.19	6.97	0	255
44	WT+Neut4h3.czi	125490.76	0.38	9.86	0	255
45	WT+Neut4h1.czi	125490.76	0.18	6.72	0	255
46	WT+Neut4h2.czi	125490.76	0.14	6.04	0	255
47	WT+Neut4h3.czi	125490.76	0.06	3.75	0	255

Red MFI measurements

1	WTLh1.czi	125490.8	0.37	9.76	0	255
2	WTLh2.czi	125490.8	20.84	69.86	0	255
3	WTLh3.czi	125490.8	4.64	34.1	0	255
4	WTLh1.czi	125490.8	6.07	38.87	0	255
5	WTLh2.czi	125490.8	5.61	37.39	0	255
6	WTLh3.czi	125490.8	5.76	37.88	0	255
7	WT+NeutL	125490.8	16.63	62.97	0	255
8	WT+NeutL	125490.8	19.06	67.06	0	255
9	WT+NeutL	125490.8	24.71	75.43	0	255
10	WT+NeutL	125490.8	4.38	33.13	0	255
11	WT+NeutL	125490.8	12.16	54.34	0	255
12	WT+NeutL	125490.8	17.6	64.63	0	255

2. **Histopathology scoring for inflammatory score**

A. Parameters considered for liver inflammation: Hepatocyte degeneration, necrosis, portal inflammation, portal fibrosis, edema, infiltration, hemorrhage, vacuolization

**0**	**Normal**	Intact lobular architecture; no inflammatory cell clusters; normal neonatal extramedullary hematopoiesis (EMH).
**1**	**Minimal**	Rare, isolated foci of inflammatory cells (neutrophils); <10% of the liver parenchyma affected.
**2**	**Mild**	Small, multifocal aggregates of neutrophils; 10–25% of parenchyma involved; mild hepatocyte swelling (degeneration).
**3**	**Moderate**	Frequent inflammatory foci; small areas of centrilobular necrosis; 26–50% of the field affected.
**4**	**Marked**	Extensive neutrophil infiltration; larger necrotic zones; possible micro-thrombi in sinusoids; 51–75% affected.
**5**	**Severe**	Coalescing necrotic lesions or abscesses; widespread hemorrhage; >75% of the liver parenchyma damaged.

B. Parameters considered for lung inflammation: Alveolar/bronchial wall thickening, structure distortion, fibrosis, alveolar congestion, hemorrhage, edema, inflammatory cell infiltration, goblet cell hyperplasia, alveolar necrosis etc.

**0**	**Normal**	No inflammatory cells; normal saccular/alveolar structure for age.
**1**	**Minimal**	Rare, scattered neutrophils in the interstitium; <10% of the lung field affected.
**2**	**Mild**	Small clusters of neutrophils; minor septal thickening; 10–25% of the field affected.
**3**	**Moderate**	Frequent neutrophil aggregates in airspaces; visible edema; 26–50% of the field affected.
**4**	**Marked**	Dense cellular infiltrates; significant septal thickening; 51–75% of the field affected.
**5**	**Severe**	Complete consolidation (loss of airspaces); widespread hemorrhage/necrosis; >75% affected.
